# GRIEF SYMPTOM PROFILES OF OLDER SPOUSES BEREAVED BY COVID-19

**DOI:** 10.1093/geroni/igad104.2051

**Published:** 2023-12-21

**Authors:** Sarah Stahl, Joseph Kazan, Robert Krafty, Charles Reynolds, Bruce Rollman, Stephen Smagula, Marie Anne Gebara

**Affiliations:** University of Pittsburgh, Pittsburgh, Pennsylvania, United States; University of Pittsburgh, Pittsburgh, Pennsylvania, United States; Emory University, Atlanta, Georgia, United States; University of Pittsrbugh, Pittsburgh, Pennsylvania, United States; University of Pittsrbugh, Pittsburgh, Pennsylvania, United States; Pitt SOM, Pittsburgh, Pennsylvania, United States; University of Pittsburgh, Pittsburgh, Pennsylvania, United States

## Abstract

Many older adults lost their spouse or life partner to COVID-19. Despite the anticipated rise in the prevalence of prolonged grief disorder, the association between COVID-19 bereavement and symptoms of complicated (or pathological) grief in community-dwelling older adults remains unclear. This study compared six clinically meaningful complicated grief symptom profiles between older spouses who were and were not bereaved by COVID-19. Participants included 80 adults aged 60+ years (20% men, 10% Black/minority status) who lost their spouse or life partner within the previous 12 months (M time since loss=6.6 months [SD=3.4 months]) and were enrolled in a randomized controlled trial for depression prevention. Grief symptom profiles were assessed with the Inventory of Complicated Grief. At the time of enrollment, older spouses bereaved by COVID-19 (n=18) reported more shock and disbelief (M = 7.11 [SD=3.16] versus M=3.83 [SD3.30], F=13.84, p<.001) and more hallucinations (M=1.56 [SD=1.29] versus M=0.45 [SD=0.88], F=17.12, p<.001) compared to older spouses bereaved by other causes of death (n=62). The two groups were similar in terms of yearning, anger and bitterness, estrangement from others, and avoidance behaviors. In addition, older spouses bereaved by COVID-19 had over 3 and a half times the odds of screening positive for probable prolonged grief disorder (OR=3.84 (95% CI = 1.28 -11.53, p = 0.016). These symptom clusters, particularly hallucinations, map to the highest level of prolonged grief disorder severity. These findings suggest that clinical treatment for prolonged grief disorder may be warranted for surviving spouses who lost their loved one to COVID-19.

